# Improvement Initiative to Ensure Quality Instrumentation in the OR

**DOI:** 10.1097/pq9.0000000000000371

**Published:** 2020-12-28

**Authors:** Renda J. Palo, Qran Dulaney Bumpers, Yasamin Mohsenian

**Affiliations:** Sterile Processing and Supply Chain, Seattle Children’s Hospital, Seattle, Wash.

## Abstract

At Seattle Children’s Hospital, in November 2016, the operating room (OR) physicians reported experiencing a high number of issues occurring during cases and believed a significant amount was related to sterile processing department (SPD) errors. These errors, hereafter called “defects,” were not defined or routinely reported. There was no method of capturing these defects. There was no root cause analysis or trending of defect data. This project aimed to improve the quality of surgical instruments received in the OR.

**Methods:**

The SPD and OR leaders collaborated to develop an OR Case Sign-Out form to capture defects during the case. The data were triaged and assigned to specific departments for root cause analysis. The SPD related data were depicted with a Pareto chart to highlight the most significant opportunities for improvement. We developed a driver diagram and identified the following interventions: orientation and competency, technician OR rotation, capacity/full-time employee analysis, surgical instruments inventory, instrument pouch work trigger, work environment, preventative maintenance, and instrument wrap reduction.

**Results:**

A 56% improvement in “Non-Sterile” defects was achieved. While a centerline shift in “Sterile” defects was not observed, the most significant “Sterile” defect, “breach of soft instrument wrap,” dropped from 8 occurrences (at baseline) to 1. The number of OR case sign-out forms collected plateaued at 47%, which could indicate missing defect data.

**Conclusions:**

SPD improved quality in the OR by reducing instrument defects. The physicians gained a mechanism for reporting barriers and tracking improvements. Ultimately, the utilization of lean tools and a quality improvement approach helped drive process changes, creating a more efficient, collaborative, and safe procedural environment for patients and staff.

## INTRODUCTION

In November 2016, the operating room (OR) physicians reported experiencing a high number of defects during cases and believed a significant amount was related to sterile processing department (SPD). Knowing that “proper sterilization of instruments used in surgical procedures is a crucial step toward reducing surgical infections, perioperative morbidity, and operative time and cost,”^1^ we had a sense of urgency to improve immediately. No method of capturing defects existed. There was no existing root cause analysis or trending of defect data.

At the beginning of the project, SPD leaders did some research on published work regarding improvements in Sterile Processing. We came across an article completed by Virginia Mason Medical Center in Seattle, WA, that used a “daily defect sheet” captured in SPD, to reduce their defects by 50%.^2^ Similarly, Seattle Children’s SPD tried collecting defect information any time the team was verbally notified of an issue. However, this method left gaps in the data and failed to capture all defects. We found that the OR staff reported only defects that seemed to be a sterilization issue, failing to report smaller issues, such as incorrect or missing instruments. We could not find any other published defect rates.

A study conducted at Zagazig University Hospital in Sharkia, Egypt, used a PDCA approach to identify and resolve SPD issues; however, it lacked a specific method for capturing defects.^3^ An article by Operational Performance Solutions, Inc. gathered data on defects by using a customer satisfaction survey.^4^ While that approach did capture defects, it was data that could be subject to opinion and memory, not real-time data. Another article referenced a reduction in total instruments within a tray to reduce costs and burden on SPD.^5^ We earmarked set utilization as a separate future project, deciding to focus on department stabilization first.

### Aim

This quality improvement project aimed to decrease the SPD defect rate by documenting data trends, analyzing root cause, and implementing improvement plans. When we achieved a baseline, the defects were delineated into non-sterile defects and sterile defects to help measure results and identify the severity of the issue reported.

1. Sterile defects included a breach in the instrument wrap, hole in the filter, hair in a set, bioburden on the instrument, etc.
2. Non-sterile defects included missing instruments, incorrect instruments, paperwork issues, etc.

We decided a reasonable improvement target would be approximately 20%–25% overall. Our final aim was: by December 2018; (1) to reduce sterile defects per 1,000 cases from 6.2 to <6.0, an improvement of 4% or greater, and (2) to reduce non-sterile defects per 1,000 cases from 46.8 to *<*38, an improvement of 19% or greater.

## METHODS

### Context

Seattle Children’s is accredited by DNV-GL and is ISO 9001:2015 certified.^6^ SPD adheres to the American National Standard ANSI/AAMI ST79:2017, a comprehensive guide to steam sterilization and sterility assurance in healthcare facilities.^7^ Both of these standards were used in validating SPD requirements during our improvement project.

Seattle Children’s Laurelhurst hospital location has 15 OR’s and averages between 40 and 60 cases per day. The SPD team employed 22 employees, over 24 hours when the action plan began. Annually, the SPD team processes 1.8M instrument sets, 80K individual instruments, and 6K lensed instruments, such as flexible scopes.

When the project began, OR staff reported issues verbally and with minimal context. They did not provide the full details of the error to SPD. SPD was not regularly called into the OR to review the defect. Daily, we spent much time trying to solve or mitigate each issue, with no trending to understand the most significant issues. The SPD team quickly realized that reacting to problems and correcting them in real-time was not an effective long term solution to preventing recurrence.

### Design

For immediate mitigation, SPD and a few surgical technicians, completed audits every day for 1 week, for all instrument sets. SPD created a quality audit checklist to ensure all sets were audited in the same way. The audit encompassed assembling the set correctly, via the component checklist, and following the job aid for preparing the container for sterilization.

It is also crucial to track and trend data over time to truly understand systemic issues.

Consequently, in collaboration with OR leadership, an “OR Case Sign-Out” form was developed to capture any defects during each OR case. This form included 4 major sections: surgical instruments, equipment and supply issues, improvement suggestions, and celebrations. The nurse in the room would complete the form. After reviewing the first day’s data, we determined that not all defects during the case were related to SPD. With department representation from SPD, Perioperative Supply, Equipment, Procedure Cards and Scheduling, and Clinical Care, we designed a process to review all OR Case Sign-Out forms during a 15-minute daily huddle. The data were triaged into ownership categories for root cause analysis.

**Fig. 1.**
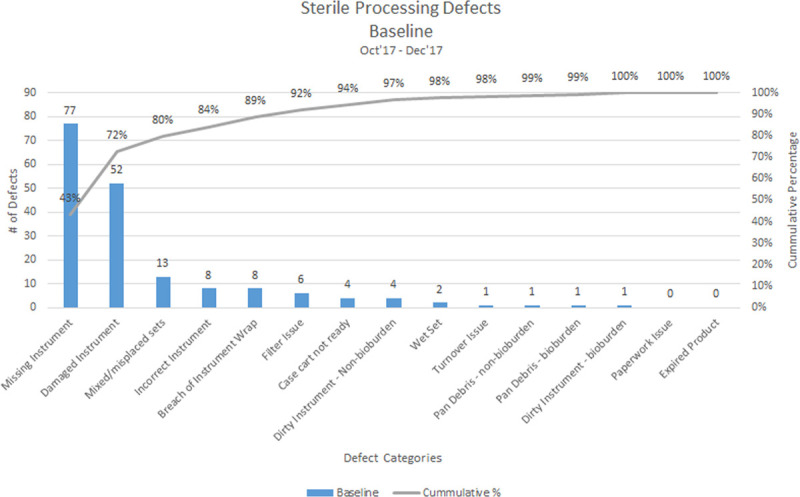
Baseline of overall sterile processing defects by defect category from October 2017 to December 2017.

**Fig. 2.**
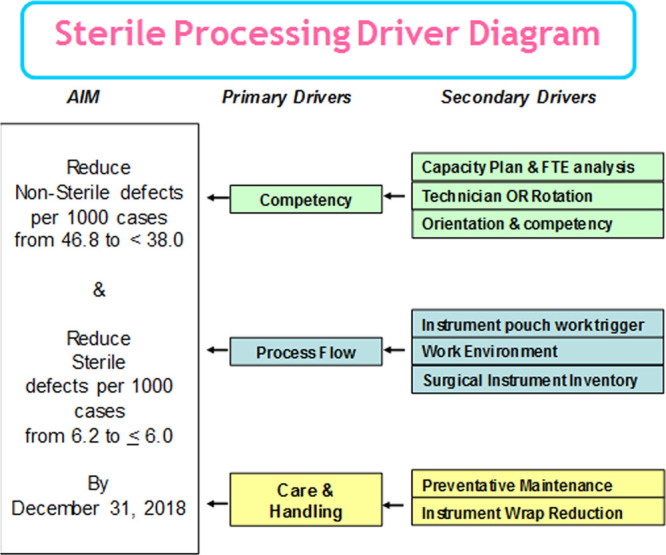
Overview of aim, primary and secondary drivers for the improvement project.

SPD developed a Pareto chart to identify the most significant opportunities for improvement (Fig. 1). The top 3 opportunities were: missing items, damaged instruments, and mixed/misplaced items within sets. We created a driver diagram to identify the action plan projects to improve (Fig. 2).

We consulted the SQUIRE guidelines in the formulation of this manuscript.^8^ The Seattle Children’s IRB reviewed this study and classified it as quality improvement and not human subject research. Therefore, IRB approval was not required.

### Improvements

#### Capacity Plan Analysis

The first step towards improvement was determining if there were enough staff to work with the highest safety and quality possible. This step was a 6-month effort that captured every step in each SPD role and the time it took to do the job correctly. Once completed, we could determine mathematically the number of staff members needed. We also had to determine the right level of leadership for the current state. SPD required leadership coverage at all times, as well as leaders for project and inventory management.

This analysis required a mid-year request for staffing to be approved by finance and executive leaders. Additionally, there was a request to finance leadership to complete a market pay analysis that ensured wages were fair for the current employees and helped hire new employees. The pay was corrected, and the hiring process of 9 new staff members began. The SPD staff increased from 22 to 31, a 30% increase, including the new positions of an Inventory Analyst and a Program Manager.

#### Orientation and Competency Assessment

As we completed the capacity plan, and new employees were onboarding, gaps in orientation became apparent. No standardized orientation existed. It was dependent upon the senior technician’s knowledge when training new employees. The assessment of competency was incomplete.

We developed a standard orientation for all new employees. All standard work, observations, and required knowledge were added to the competency assessment. Proper orientation and competency assessment would aid in reducing overall defects.

#### Technician OR Rotation

As part of the standard orientation, we added a rotation in the OR for SPD technicians. This addition helped SPD technicians to understand how instruments are to be assembled on the field, used during the procedure, flushed, and prepared for decontamination. The surgical technicians also began to rotate in SPD. SPD was able to show the OR technicians the importance of proper pre-cleaning and care and handling to prevent instrument damage and facilitate throughput. SPD and OR leaders developed a hospital policy outlining the instrument condition and transport requirements from the OR to SPD. We were able to begin tracking deviations to this policy. The defects included dirty or lack of pre-cleaning, the potential to damage instruments, mixed/misplaced instrument sets, etc. We reported to the OR in real-time, and the OR improved the condition of transport to SPD by 50% over 1 year. This change aided in reducing all 3 of our top defects: missing instruments, damaged instruments, and mixed/misplaced instruments.

#### Work Environment

Distractions during critical SPD functions can cause defects, such as having sets with missing items. The team determined that assembly and decontamination areas should be considered ‘distraction-free zones.’ We developed float roles in the assembly and decontamination areas to field phone calls and manage visitors. A process was created to give the staff freedom to wear noise-canceling headphones or listen to music using one earbud if it facilitated concentration.

We determined that some facility changes to space could ease unnecessary traffic, reduce waste, and aid in the process flow. An automatic door with SPD-only badge access was added to the decontamination area. We added a decontamination sink, replaced 2 existing sinks with 3-compartment sinks, and added an ultrasonic unit.

#### Preventative Maintenance for Surgical Instruments

Instrument damage was the #2 defect in the Pareto chart. After evaluating the repair and preventative maintenance (PM) process, we determined that the vendor was not addressing many of our inventory repair requirements. We terminated that vendor and completed a competitive bid process in search of a new vendor for instrument repair services. The new vendor did a complete physical inventory and set a PM rotation schedule based on usage. The new vendor performed 3 times per week and provided an analysis of the repairs. We altered the budget, understanding that there would be an increase in surgical instrument purchases to replace instruments that had been neglected for years. It took a year to cycle through the complete inventory.

The vendor provided education for the SPD and OR staff on instrument inspection and testing and care and handling of surgical instruments and flexible scopes. The OR to SPD defect reduction of 50%, the education in care and handling, and the skilled PM service, reduced our repair defects by 80%. Our repair costs have reduced by 10% from the previous year.

#### Surgical Instruments Inventory

The inventory in SPD was not managed appropriately. Racks and carts needed repair and were difficult to open. There were pegboards with hanging surgical instruments, hindering the “first in, first out” approach. There were no par levels or inventory locations. The team spent a considerable amount of time searching for instruments to complete the assembly of sets.

New, closed storage carts were purchased to store inventory. Inventory locations and par levels were created and added to the instrument management system, informing the assembler where to pick the inventory. To maintain these improvements, we added an Inventory Analyst role to the team. Set assembly times, for 3 service lines, reduced by 35%.

#### Soft Instrument Wrap Reduction

The top 3 items on the defect Pareto chart were classified as “Non-sterile” defects. We acknowledged that “Sterile” defects also needed to be addressed. The most significant contributor to “Sterile” defects was “breach of the soft instrument wrap.” The wrapped items were stored on metro rack shelving units. The items were handled multiple times, and the wrap itself was easy to tear.

It took 6 months to take a physical inventory of items in instrument wrap and ensure the vendor validated each set to be processed in a rigid container.^9^ We had to consider the size of the sets and space available for inventory. When the rigid containers arrived, the sets had to be reassembled in the new rigid container and denoted in the inventory. All remaining soft instrument wrapped items had either a solid tray or shelf liner underneath to prevent tears when removing or restocking. The items in soft instrument wrap reduced from 336 to 38, and defects dropped from 8 (at baseline) to 1, an 87.5% improvement.

#### Instrument Pouch (Peel Pack) Work Trigger

Some individual instruments are packaged and sterilized in a plastic-paper pouch (commonly called peel packs) for quick access when needed. Peel packs give the physician flexibility to open only one instrument, rather than an entire set. However, it was easy to misplace or mix these instruments with other used instrument sets to be cleaned in SPD. There was no apparent work trigger to know when a peel pack was used and/or needed restocking. We organized peel packs by service and unevenly distributed throughout the OR inventory core location. To manage the inventory, it required physical inventory counts daily.

SPD decided to consolidate all peel packs into 6 metro-racks to be located in the center of the Inventory core. Using a Toyota lean thinking approach, we established a 2-bin, Kanban system with set par levels.^10^ The peel packs were set up in the inventory management system as small sets to be scanned and tracked. When an empty bin is returned to SPD, peel packs are completed and restocked within 24 hours.

## RESULTS

**Fig. 3.**
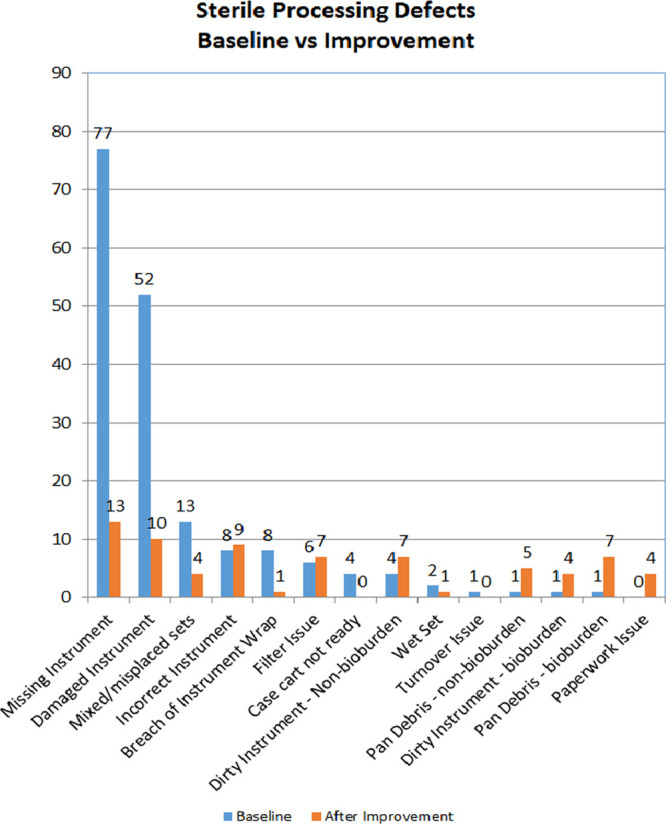
A side by side comparison by Sterile processing defect category, baseline and after quality improvement.

**Fig. 4.**
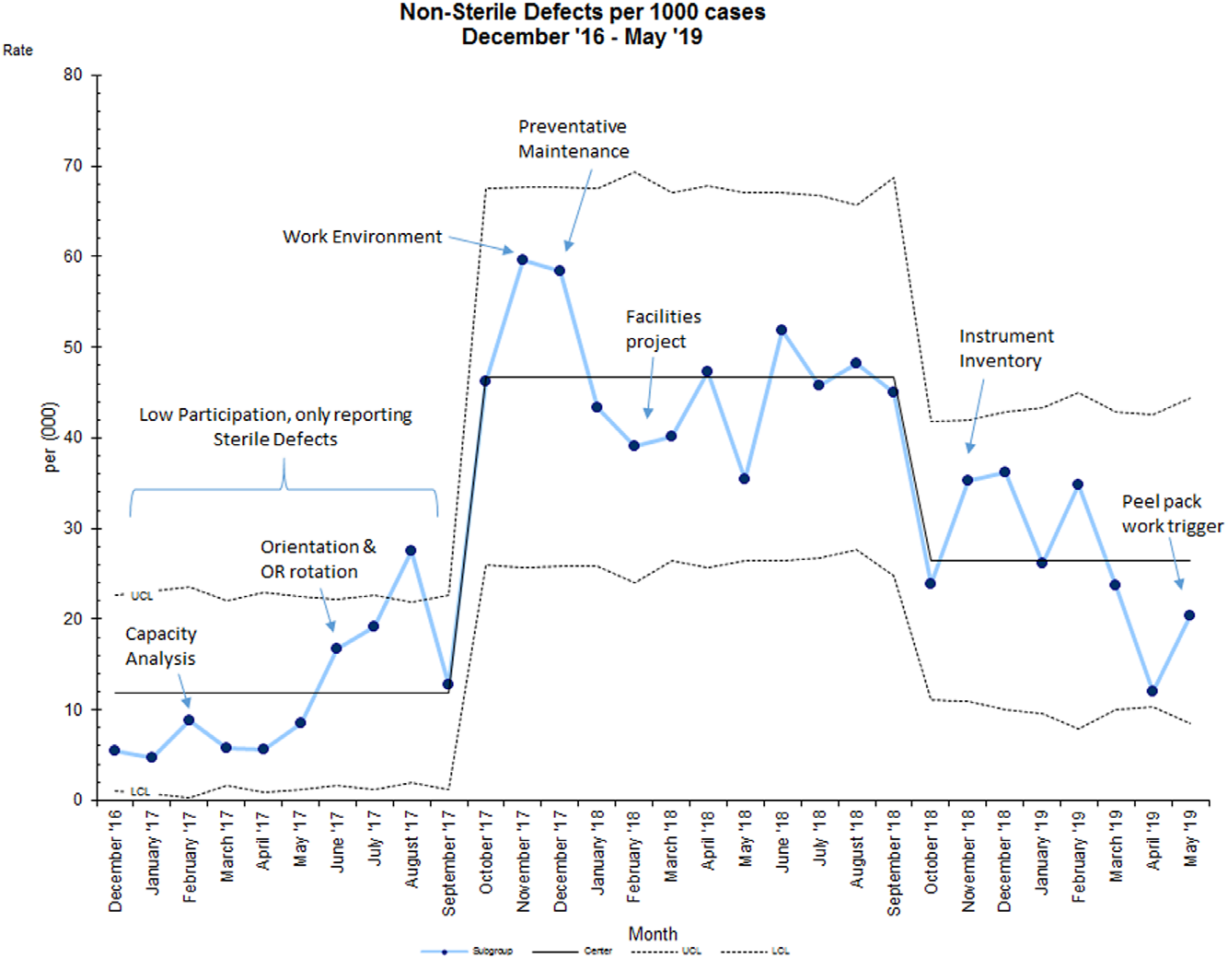
Sterile processing “Non-sterile” defects run u-chart December 2016 to May 2019.

The side by side comparison per defect category is illustrated in Figure 3. It depicts the greatest reduction in the top 3 defects. ‘Non-Sterile’ defects per 1,000 cases reduced from 46.8 to 26.5, a 56% improvement (Fig. 4). Our first data shift was due to an increase in OR case sign-out forms. With better reporting, our percentage of OR case sign out forms received increased from 26% to 47%, but quickly plateaued. This plateau could mean the remaining cases didn’t have any defects, or that defect data wasn’t captured. Our second data shift was the result of the PM, work environment, and facilities projects coming to an end, culminating in a reduction of defects.

**Fig. 5.**
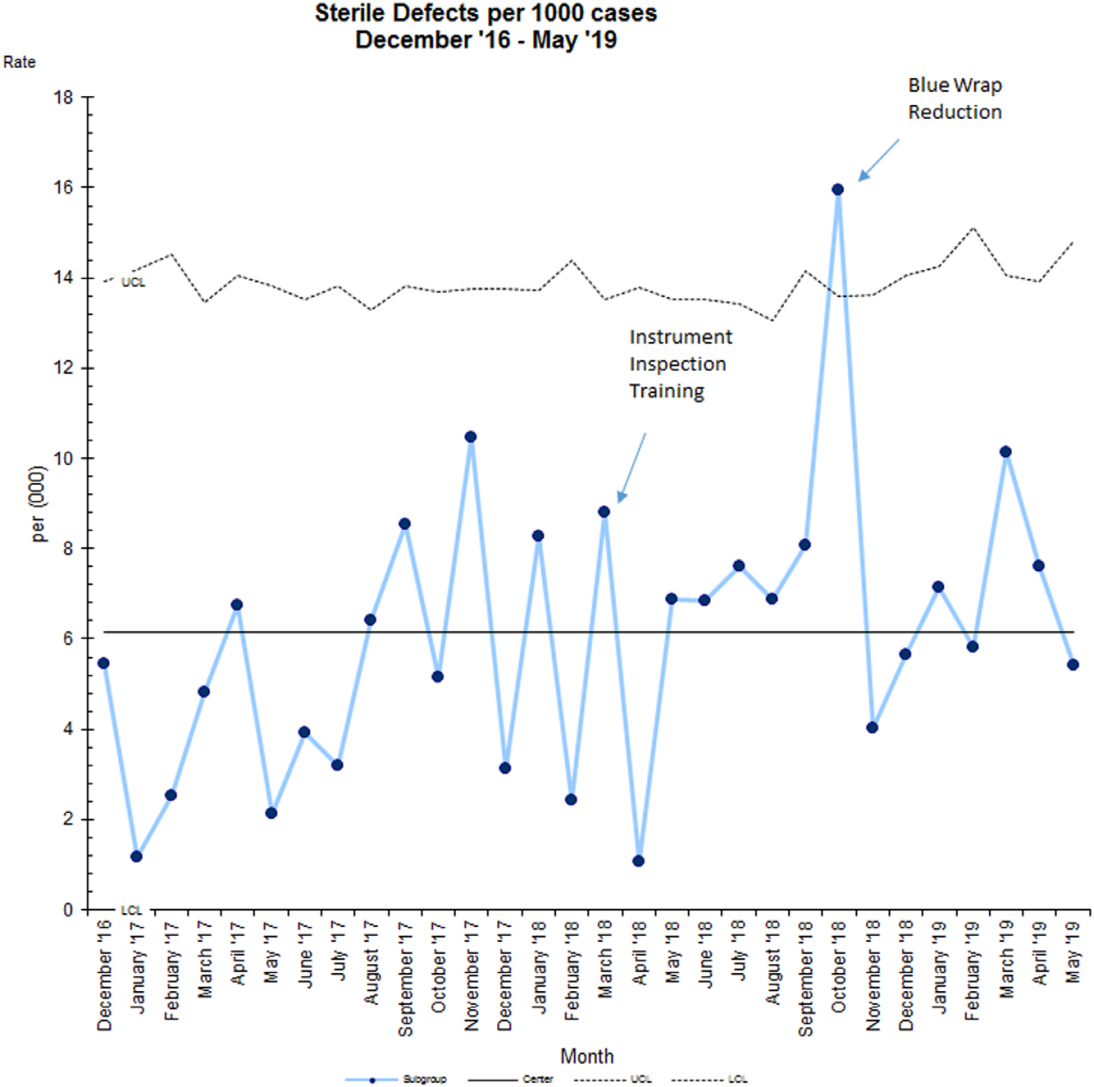
Sterile processing “Sterile” defects run u-chart December 2016 to May 2019.

“Sterile” defects per 1,000 cases did not have a significant shift in data (Fig. 5). The fact that the first sterile defect “breach of instrument wrap” was the fifth defect on the Pareto chart was a contributing factor. The team focused on the top 3 defects first, all non-sterile defects. Once we finally tackled “breach of instrument wrap,” we decreased the number of occurrences from 8 (at baseline) to 1 (after improvement). Unfortunately, while focusing on instrument wrap, the other “Sterile” defects increased slightly, as shown in Figure 3.

## DISCUSSION

Overall, SPD was able to improve quality in the OR by reducing surgical instrument defects during procedures. The physicians have a mechanism for reporting and a way to review progress on specific challenges. By utilizing lean tools and a replicable quality improvement model, we developed a more efficient, collaborative, and safe procedural environment for patients and staff. This experience has introduced lean thinking to SPD and proven effective in a healthcare setting. Sustainability of these improvements continues with customer-centric, quality-driven leadership, and a safety-first culture.

Initially, we planned to correlate a decrease in defects with either a decrease in surgical infections or in OR time cost savings. However, there are so many variables that affect infections and costs that it was difficult to say our improvements made the difference definitively. Spending time correcting defects is wasteful. Colloquially, we like to say every minute wasted in the OR equates to $100. SPD and OR leaders decided that measuring a reduction in “defects” was a straightforward way to quantify the improvement. We even chose the word “defects” purposefully. Using “contaminations” implied that every issue was “bioburden.” Using “errors” had a negative connotation to the front line staff.

One limitation of this study was the OR case sign-out form completion rate. When this project began, the percentage of forms completed per case was at 26%. The tactics below facilitated the completion of forms:

1. A physician champion is imperative to engage other physicians, communicate the importance of defect reporting, and share the resulting improvements. Our physician champion is a surgeon and the Chief of Orthopedics and Sports Medicine. She encourages other surgeons to complete the OR sign-out form and shares best practice using the form throughout the case. Many physicians do not read all “update emails” and, due to scheduling conflicts, cannot attend report out sessions. We settled on a dashboard model so that with a glance, teams could see the progress and status of action items through various graphs and tables.
2. The OR case sign-out form is located in the patient chart with other required paperwork.
3. The surgical RN (or designee) is ultimately responsible for ensuring the OR case sign-out form completion.

A large amount of time was spent coaching staff on what information required listing on the OR case sign out forms for proper root cause analysis. It also took time to explain that all defects should be listed, even if the defects were corrected immediately. It took a full year of education and collaboration to arrive at the data baseline. To encourage the completion of the OR case sign-out form, SPD leadership attends and reports improvement data in various quality meetings, including the Perioperative Service Quality Review, Perioperative Operations Council, and Division Chief’s leadership meetings.

Even after this work, the submission of OR Case Sign-out forms is approximately 47%, which is unacceptable and could mean we are missing valuable data. Steps will be taken to increase the number of OR case sign out forms completed. Ideas we are considering are (1) requiring completion in the Time-out process at the end of the case, (2) providing additional locations to turn in completed OR case sign out forms, and (3) facilitating a regular monthly meeting with all surgical RN’s to discuss and gain feedback on the percentage of OR case sign out forms completed.

Another limitation of this study was our lag in prioritizing “Sterile” defects. We spent much time tackling our top 3 defects in the Pareto chart, all non-sterile defects. Sterile defects, such as bioburden on an instrument or breach of instrument wrap, are more impactful in the OR, requiring a breakdown of the sterile field, in some cases, causing case delay and inefficient use of OR time. We learned a valuable lesson to look at the impact, not just overall occurrences when evaluating opportunities for improvement. Our root cause analysis should have considered a cost-benefit analysis of improvement, such as a Priority-Payoff matrix.^11^

Gathering data and reporting results can be disheartening if the data are unfavorable. The leadership team must remain positive, respectful, and create a safety-first atmosphere of reporting defects. Reports should be made a priority without placing blame. The front line staff must be involved in ideas for improvement and change. A cross-functional team triaging defects helps remove bias from the process. Not having all of the facts for the defect can cause a conflict on which department owns the defect, thus skewing the data. As these conflicts arise, it is helpful to document the decision and revisit it later if needed.

Sustainability requires an engaged, collaborative, quality-focused team. The next steps include exploring additional double checks of the instrument set before sterilization and working with our physicians on set contents. We will continue to make the collection of defects as easy as possible. Now that we have built a sustainable quality program in SPD, we can focus next on improving efficiency and increasing throughput.

## CONCLUDING SUMMARY

Ultimately, SPD improved quality in the OR by reducing instrument defects. The physicians gained a mechanism for reporting barriers and tracking improvements. The utilization of lean tools and a quality improvement approach helped drive process changes, creating a more efficient, collaborative, and safe procedural environment for patients and staff.

## DISCLOSURE

The authors have no financial interest to declare in relation to the content of this article.
